# Cancer pain: molecular mechanisms and management

**DOI:** 10.1186/s43556-025-00289-0

**Published:** 2025-06-28

**Authors:** Wan-Li Wang, Yi-Hang Hao, Xin Pang, Ya-Ling Tang

**Affiliations:** 1 State Key Laboratory of Oral Diseases & National Clinical Research Center for Oral Diseases & Department of Oral and Maxillofacial Surgery, West China Hospital of Stomatology, Sichuan University, Chengdu, China; 2 State Key Laboratory of Oral Diseases & National Clinical Research Center for Oral Diseases & Department of Temporomandibular Joint, West China Hospital of Stomatology, Sichuan University, Chengdu, China; 3 State Key Laboratory of Oral Diseases & National Clinical Research Center for Oral Diseases & Department of Oral Pathology, West China Hospital of Stomatology, Sichuan University, Chengdu, China

**Keywords:** Cancer pain, Molecular mechanism, Assessment, Treatment

## Abstract

Cancer pain, a highly prevalent and distressing symptom among cancer patients, has a seriously harmful effect on their life and presents a complex challenge in clinical management. Despite extensive research efforts and the existence of clinical guidelines, significant controversies persist regarding the molecular mechanisms underpinning cancer pain as well as the most effective management strategies. This review systematically delves into the neurobiological underpinnings of cancer pain, centering on the interplay of peripheral and central sensitization, cellular stress and dysfunction, as well as the crucial roles of various signaling pathways and epigenetic regulation in its pathogenesis. In terms of treatment, the fundamental strategy involves a comprehensive initial assessment of cancer pain, followed by targeted interventions based on the assessment findings. It advocates for a multimodal approach that integrates pharmacological with non–pharmacological therapies. However, ongoing debates surround issues related to opioid rotation protocols and the long-term safety of opioid use. Furthermore, it underscores the underexplored potential of personalized therapies targeting molecular pathways and the need for standardized, interdisciplinary pain assessment tools. By bridging mechanistic research and clinical practice, this work potentially provides a framework for refining guideline implementation, advancing targeted therapies, and improving patient-centered care, thereby contributing to the evolution of precision oncology and holistic pain management paradigms.

## Introduction

In 2022, approximately 20 million new cancer cases were diagnosed globally, and cancer-related deaths reached 9.7 million. Projections suggest that by 2050, the global incidence of new cancer cases will surpass 35 million. Studies show that more than 70% of cancer patients experience pain at some point, with about 90% of those with advanced cancer reporting pain [1]. Cancer-related pain significantly increases physical suffering and severely affects mental health and daily quality of life, further exacerbating the burden on patients. Furthermore, cancer pain can complicate treatment and recovery, potentially reducing patients’ adherence to therapy [2, 3].

**Fig. 1:**
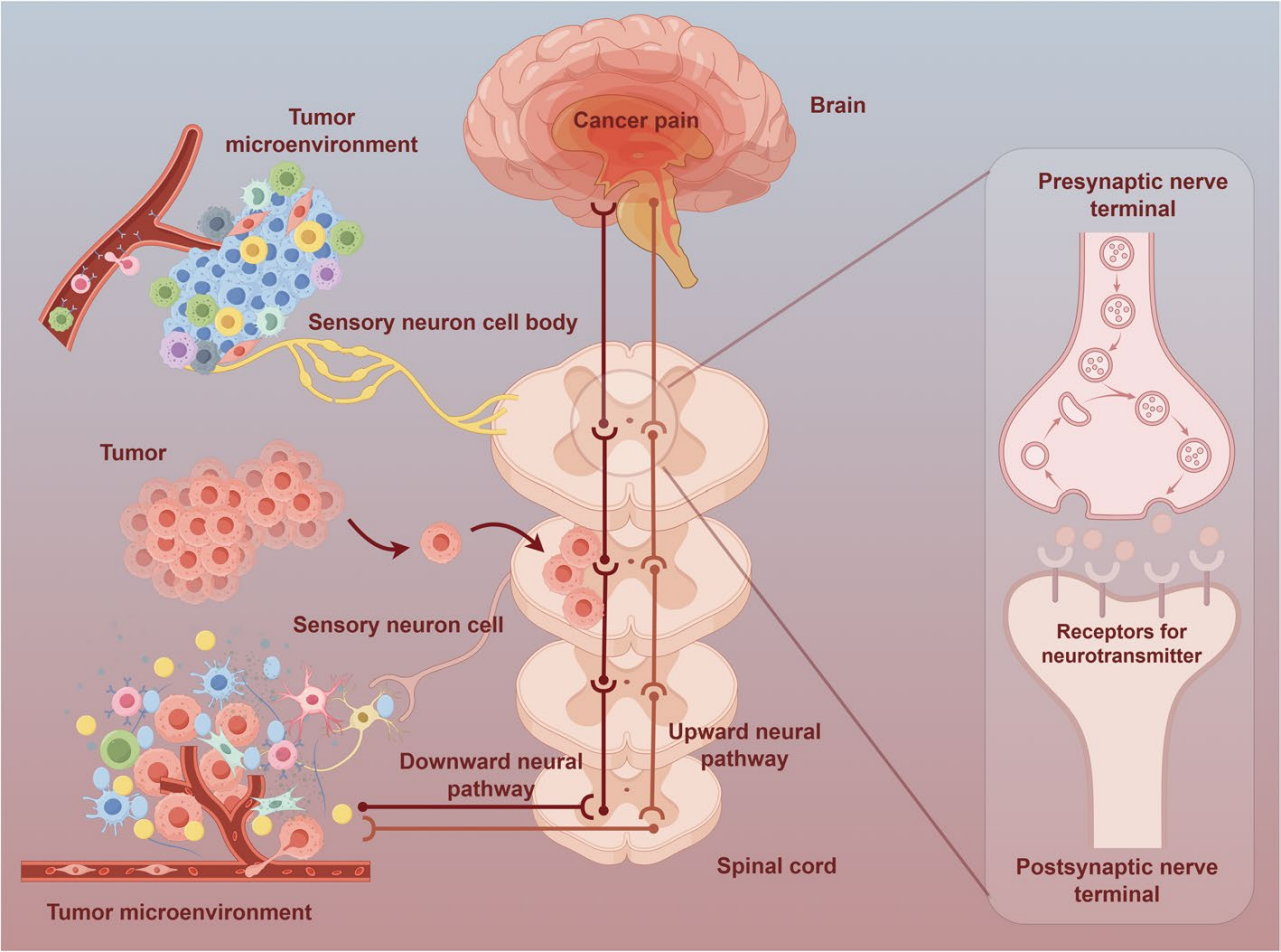
Cancer pain signaling pathway. Within the tumor microenvironment, sensory neurons expressing specific receptors detect noxious stimuli released by cancer and immune cells, initiating pain signals. The cell bodies and axons of these neurons serve as vital for sending signals to the spinal cord. When signals reach the spinal cord, specialized neurons not only relay them to higher brain regions but also participate in complex local circuit interactions. The spinal cord contains various modulatory mechanisms mediated by neurotransmitters and receptors. These mechanisms can amplify or dampen pain signals depending on contextual and regulatory factors. At presynaptic terminals, neurotransmitters are precisely released to transmit signals to postsynaptic neurons. Postsynaptic terminals equipped with specific neurotransmitter receptors ensure efficient signal reception and neural activation. These signals then ascend to the brain, where complex processing occurs in regions including the thalamus and cerebral cortex. The downward regulation of cancer pain is mainly driven by the endogenous analgesic system of the brainstem. The endogenous analgesic system inhibits cancer pain transmission by releasing opioids and neurotransmitters like norepinephrine and serotonin, which activate receptors in the spinal cord and brainstem. Endogenous opioids inhibit pain transmission by binding to μ-opioid receptors in the spinal cord dorsal horn, reducing neurotransmitter release

The International Association for the Study of Pain (IASP) characterizes chronic cancer-related pain as persistent discomfort originating either from primary malignancies or metastatic spread, and alternatively arising as a consequence of therapeutic interventions [4]. What’s more, the observed pain manifestations require differentiation from concurrent symptoms attributable to comorbid pathological conditions. It is the body’s response to harmful stimuli, involving both physiological and psychological components and complex neurobiological processes. The transmission mechanism of cancer pain involves complex upward and downward neural pathways (Fig. 1).

It develops through complex biological processes, including tumor cell invasion of nerve tissue, immune system activation, and nervous system remodeling [5–7]. Continued exploration of cancer pain’s pathophysiological mechanisms promises to elucidate the molecular underpinnings of nociceptive pathway dysregulation, aberrant signal transduction and maladaptive neuroplasticity, while also opening new avenues for drug development targeting key pain-related molecules. Future research should prioritize exploring these biological foundations, especially how the tumor microenvironment impacts pain onset and persistence. Concurrently, research should emphasize identifying new targets and therapeutic strategies to develop safer and more effective treatments, thus alleviating cancer patients’ pain.

To enhance the quality of cancer pain management, professional organizations and academic institutions worldwide have published or updated various clinical guidelines. These guidelines aim to standardize the diagnosis and treatment of cancer pain, improve nursing quality in clinical practice, and enhance patients’ quality of life. However, significant differences exist between the guidelines, particularly regarding the quality of evidence, recommended treatments, and applicable clinical scenarios [8–10]. This variability creates challenges for clinicians, particularly when selecting the most appropriate treatment. This review summarizes and analyzes the current guidelines for cancer pain management. We have selected 7 guidelines to systematically and critically review cancer pain management (Table 1). Our goal is to provide clearer, evidence-based references for future clinical decisions while addressing the challenges and limitations of guideline implementation in practice. Additionally, we provide suggestions for future research, with the aim of contributing to the advancement of pain management.

**Table 1:** The basic information of guidelines

Organization	Year	Region/Country	Evidence-based evidence	Population	Ref
ASCO	2022	America	Based on randomized controlled trials and observational studies	It is suitable for adult cancer patients and is not recommended for pediatric patients	[11]
CCO	2017	Canada	Based on Canadian epidemiological research and clinical practice	It is suitable for oncologists and cancer patients	[12]
INCMNSZ	2017	Latin America	Based on the Mexican patient cohort study	It is mainly applicable to adult cancer patients	[13]
ESMO	2018	Switzerland	Based on randomized controlled trials and meta-analysis	It is mainly applicable to adult cancer patients	[14]
WHO	2018	Switzerland	Based on a large amount of clinical experience and research	It is suitable for all cancer patients with pain, especially those with moderate to severe pain	[15]
JSPM	2022	Japan	Based on expert consensus	It is mainly targeted at cancer patients undergoing chemotherapy	[16]
NCCN	2025	America	Based on systematic evaluation and expert consensus	It is mainly applicable to adult cancer patients	[17]

*ACSO* American Society of Clinical Oncology, *CCO* Cancer Care Ontario, *INCMNSZ* National Institute of Medical Sciences and Nutrition Salvador Zubirán, *ESMO* European Society for Medical Oncology, *WHO* World Health Organization, *JSPM* Japan Society for Palliative Medicine, *NCCN* National Comprehensive Cancer Network

## Causes of cancer pain

**Fig. 2:**
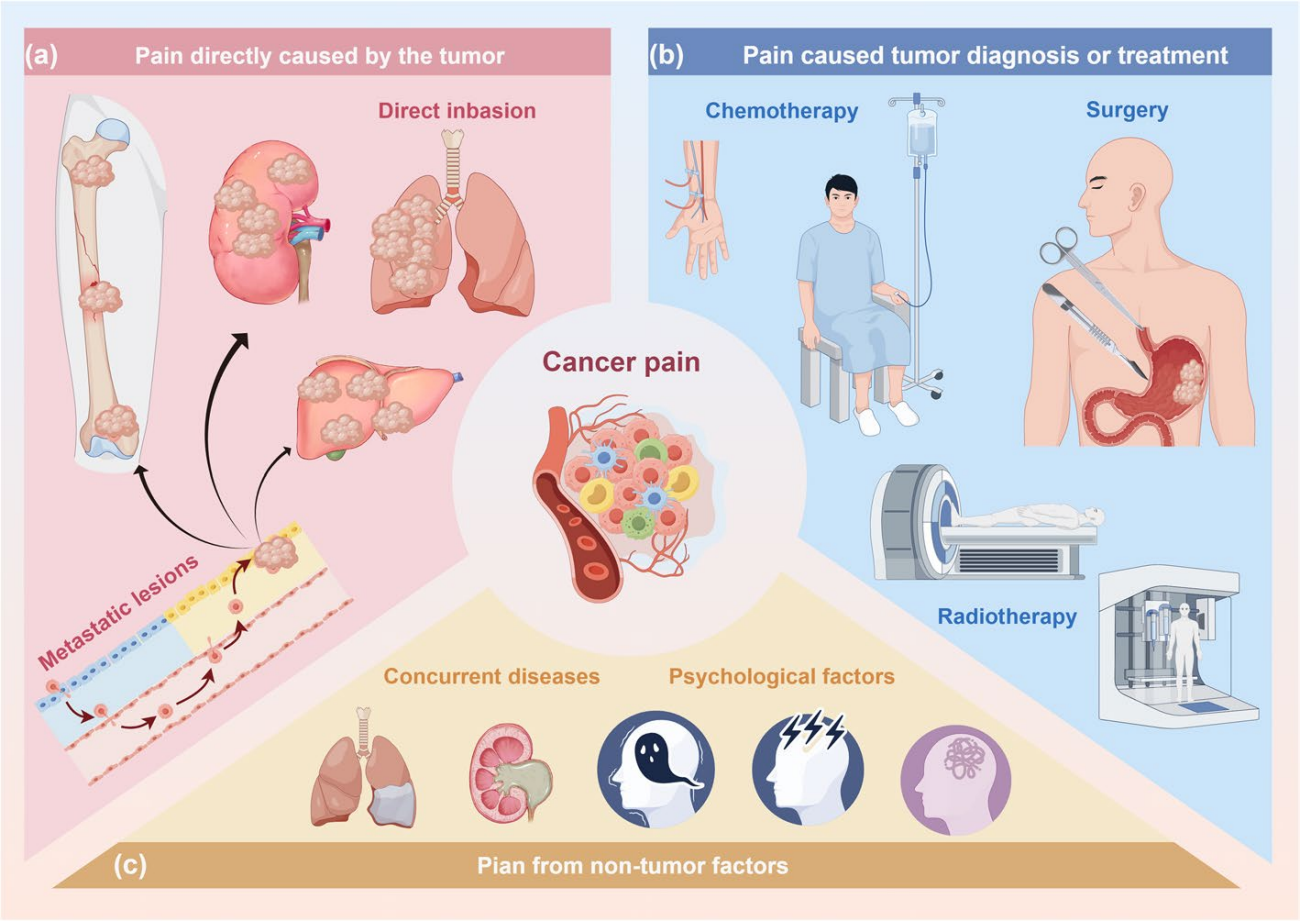
Etiological classification of cancer pain. **a** Tumor-direct effects: direct invasion (tumor invades and damages bones, nerves, or organs); metastatic lesions (metastases in critical organs (brain, liver, bone) through tissue infiltration and destruction); occupying effect (tumor growth exerts compressive, obstructive, or ischemic effects on surrounding tissues); paraneoplastic syndromes (systemic effects of tumors, such as abnormal hormone secretion or immune reactions, contribute to pain perception). **b** Diagnosis and treatment effects: surgery (postoperative incisional pain and nerve damage from surgical procedures); chemotherapy (chemotherapy-induced peripheral neuropathy and mucositis); radiotherapy (radiation-induced fibrosis and brachial or lumbar plexus lesions). **c** Non-tumor factors: psychological factors (anxiety and depression exacerbate pain perception through neuroendocrine and immune modulation); concurrent diseases (conditions like diabetic neuropathy interact with cancer pain, complicating management)

It is a common clinical symptom in cancer patients and significantly impacts their quality of life. Cancer pain could be stratified into 3 distinct types: pain directly caused by the tumor, pain resulting from cancer diagnosis or treatment, and pain from non-cancer-related factors [18–21] (Fig. 2).

### Pain directly caused by tumor

Cancer pain is typically caused by tumor growth, spread, and metastasis. Tumor cells not only invade local tissues but may also compress surrounding structures and invade nerves or blood vessels, causing ischemia, necrosis, or nerve damage, which leads to pain [22, 23]. For example, lung cancer patients may experience severe chest pain from tumor compression of the chest wall or intercostal nerves [24, 25]. Brain tumors can cause headaches and neuralgia by compressing brain tissue [26], while nasopharyngeal cancer with cervical metastasis may compress the brachial nerve plexus, inducing radicular pain patterns along corresponding dermatomal distributions [27, 28]. Retroperitoneal tumors compressing the lumbar or abdominal nerve plexus may cause lower back or abdominal pain [29, 30]. This pain is often characterized by a persistent dull ache, accompanied by tingling or a burning sensation. Cancer pain is also closely associated with the invasion of bone tissue by tumor cells. Tumor cells affect osteoblasts, osteoclasts, and nerve fiber effectors within the bone, promoting bone resorption, weakening bone integrity, and causing mechanical deformation or increased intraosseous pressure. This pathological bone remodeling causes bone pain and increases the risk of pathological fractures [6, 31, 32]. Tumor tissue can also cause dysfunction in other organs, indirectly triggering pain. For example, cervical cancer pressing on the urinary tract can cause hydronephrosis and severe back pain, while gastrointestinal obstruction may result in intense abdominal pain and colic [33, 34]. Tumor growth may also cause local tissue necrosis or infection, leading to inflammatory responses and exacerbating pain. For example, tumor-induced bowel obstruction, gastric bleeding, or liver tumors causing liver necrosis may result in acute abdominal pain and systemic inflammation [35, 36].

### Pain caused tumor diagnosis or treatment

In the cancer diagnostic process, some invasive procedures may cause pain [37, 38]. Tissue biopsy, the gold standard for cancer diagnosis, involves inserting a needle or surgically removing tissue for pathological analysis, potentially bringing local tissue damage and inflammation, thus leading to pain [39]. Some imaging procedures, like Positron emission tomography/Computed tomography (PET/CT) scans, don’t directly cause pain but may require patients to hold uncomfortable positions, leading to discomfort or pain. Various therapeutic modalities during cancer treatment can also trigger pain.

Surgery is a common treatment for cancer, but postoperative pain remains a prevalent clinical issue, typically categorized into acute and chronic types [40]. Acute postoperative pain typically manifests within hours to days after surgery, often resulting from tissue damage during tumor removal, cutting, or dissection. Surgical procedures may also affect vital organs or structures, causing inflammatory responses and pain [41–43]. Chronic postoperative pain is frequently linked to nerve damage, scar formation, and other complications. Unintentional nerve damage during surgery, including injury to the phrenic or peripheral nerves, can result in chronic neuropathic pain [44, 45]. Breast cancer patients undergoing mastectomy may experience “phantom limb” pain or persistent pain at the surgical site due to nerve damage [46, 47].

Chemotherapy-induced peripheral neuropathy (CIPN) is characterized by chronic pain resulting from peripheral nerve damage caused by chemotherapy. Common symptoms include numbness, stabbing pain, and burning sensations in the limbs [48–50]. It may present acutely during treatment, with gradual recovery after chemotherapy or develop after treatment ends [51]. CIPN incidence is highest following treatment with platinum-based drugs, paclitaxel, or vinca alkaloids. The incidence of CIPN caused by paclitaxel ranges from 61 to 92%, while that caused by vinca alkaloids is around 20%. The incidence of CIPN with platinum-based drugs ranges from 12 to 85%, and for oxaliplatin, acute incidence ranges from 85 to 96%, while chronic incidence ranges from 40 to 93% [51–55]. A meta-analysis of 31 cohort studies involving 4,179 cancer patients found the incidence rates of acute and chronic CIPN to be 89% and 85%, respectively [51].

Radiotherapeutic interventions employ ionizing radiation beams to induce lethal DNA damage in neoplastic cells through targeted energy deposition, but they may also damage surrounding healthy tissues. Patients undergoing radiation therapy frequently report experiencing varying degrees of pain during treatment cycles. Around 85% of cancer patients receiving radiotherapy experience pain and burning sensations, with some developing chronic pain in the irradiated area [56, 57]. Radiotherapy regimens for head and neck cancer patients are associated with treatment-emergent orofacial pain in 23–27% of cases [58, 59]. Among breast cancer patients receiving radiotherapy, 23.2% report mild pain, and 9% experience moderate to severe pain. The risk of pain increases with higher radiation doses or larger treatment areas [60]. The type and severity of pain also depend on the treatment area. For example, chest radiotherapy may cause pleuritis, abdominal radiotherapy may result in gastrointestinal discomfort and abdominal pain, and pelvic radiotherapy may cause pain in the reproductive organs, intestines, or bones [61–64].

### Pain from non-tumor factors

Besides tumor-related pain from the disease, diagnosis, and treatment, cancer patients may also suffer from pain due to non-tumor factors. These factors include comorbidities, complications, and psychosocial issues. Comorbid conditions substantially modulate pain perception dynamics in oncology populations. Pre-existing chronic pathophysiological states (diabetic neuropathy, cardiometabolic disorders, degenerative joint pathologies) establish neural sensitization pathways that synergize with cancer-related nociceptive signaling [21, 65]. These conditions may be related to cancer treatment, either directly or indirectly, or they may exist independently. For example, diabetic patients may experience pain from diabetic neuropathy [66, 67]. Throughout their illness, cancer patients often experience significant psychological stress, including anxiety and depression [68]. These psychological factors can worsen pain perception, creating a vicious cycle. Anxiety and depression increase pain sensitivity, causing patients to focus more on their discomfort and worry, which intensifies the pain experience.

## Types of cancer pain

Cancer pain, a highly intricate and multifaceted issue, can be categorized into distinct types based on its pathophysiological mechanisms as well as the duration of its occurrence. These classifications are of paramount importance for the effective therapeutic navigation of cancer pain.

### Nociceptive pain and neuropathic pain

Based on the pathophysiological mechanisms of cancer pain, it can generally be categorized into two types: nociceptive pain and neuropathic pain. These two types of pain not only differ in their clinical presentation but also exhibit significant differences in their underlying mechanisms [19, 69]. As the predominant subtype of cancer pain, nociceptive pain manifests through distinct pathophysiological pathways. Current classification stratifies this pain modality into visceral and somatic nociception. The former, classified by its anatomical origin, can be categorized as thoracic, abdominal, or pelvic pain [70–72]. Neuro-physiologically, visceral pain can be classified as either true visceral pain or referred pain. The primary causes of visceral pain include tumor invasion of hollow organs (gastrointestinal tract, bile ducts, bladder), nerve involvement, release of pain-inducing substances, tumor-induced ischemia, or pain from cancer treatments. Visceral pain is often deep, dull, and poorly localized, with a diffuse quality [73–75]. This pain can radiate to other parts of the body, such as lumbar and inguinal pain from kidney tumors or right shoulder pain from liver or gallbladder tumors [76, 77]. Somatic cancer pain can be classified into superficial and deep types. Somatic pain is associated with body tissues, including skin, muscles, and bones. It can be further divided into superficial and deep somatic pain. Clinically, somatic pain is characterized by localized dull, aching, throbbing, or sharp sensations [78, 79]. Bone cancer pain is a common and intense form of somatic pain. Bone cancer pain often results from tumor-induced bone metastasis, nerve infiltration, bone stretching or compression, bone destruction, mechanical instability, and increased intraosseous pressure [31, 80].

Oncologic neuropathic pain originates from structural impairment of somatosensory pathways, which may be triggered by the tumor itself or emerge as a result of cancer therapies. It is commonly linked to tumor compression or nerve infiltration. The cancer treatments could result in neural injury, which in turn may lead to neuropathic pain [81, 82]. Cancer-related neuropathic pain is characterized by spontaneous pain, localized pain at the site of nerve injury, and frequently, sympathetic nerve abnormalities. Clinically, it presents as burning, electric shock-like, shooting, stabbing, lightning-like, or numbness-like pain [83].

### Acute pian and chronic pain

Cancer pain can be categorized into acute and chronic pain based on its duration [84, 85]. Acute pain is intense, sudden, and short-lasting, often triggered by specific events or stimuli, such as surgery, radiotherapy, or chemotherapy. It is characterized by its intensity, sudden onset, and sharpness, gradually alleviating once the underlying cause or treatment is addressed. Chronic pain, however, persists for a longer duration, usually lasting more than three months. It may be intermittent or persistent. In cancer patients, chronic pain is often associated with tumor growth, metastasis, or long-term treatment-related damage. Chronic pain is typically more complex and harder to fully resolve. However, with ongoing management and treatment, patients can often achieve some relief.

### Cancer breakthrough pain

Cancer breakthrough pain is a unique form of cancer-related pain. It refers to sudden, intense pain in cancer patients who are undergoing adequate pain management and maintaining relatively stable pain control. This pain may occur spontaneously or be triggered by predictable or unpredictable factors [86–88]. Breakthrough pain is classified into two types: incident (triggered) pain and spontaneous pain. Incident breakthrough pain is typically triggered by specific factors, such as increased pain during movement in patients with bone metastases. Spontaneous breakthrough pain occurs without specific triggers, often exacerbating conditions like neuropathic pain [89]. Breakthrough pain typically has a rapid onset, reaching peak intensity within minutes, and lasts no longer than 30–60 min. It occurs at least once daily and is typically of severe intensity. Patients with visceral pain, bone cancer pain, or neuropathic pain may experience breakthrough pain. The occurrence of breakthrough pain often indicates tumor progression and more severe bodily damage [90].

## Molecular mechanisms of cancer pain

Cancer pain’s molecular mechanisms are intricate, stemming from multiple factors’ interplay. Inflammatory mediators play a crucial role in regulating immune responses, neuronal excitability, and pain signaling pathways. Neurotransmitters modulate pain perception, while ion channels affect the electrophysiological properties of neurons. Furthermore, endoplasmic reticulum stress plays a role in regulating pain signal transmission. The combined effects of these elements lead to both the initiation and sustained progression of cancer-related pain. Targeting these molecular mechanisms with therapeutic strategies may offer new approaches to alleviate cancer pain.

### Peripheral sensitization

#### Inflammatory mediators

Belonging to the cytokine family, interleukin-6 (IL-6) serves as a molecular switch initiating and sustaining inflammatory signaling pathways, and has been shown to amplify the production of other inflammatory mediators. Studies have demonstrated that IL-6 could enhance the production of tumor necrosis factor-α (TNF-α) and interleukin-1beta (IL-1β), which are also critical in pain signaling pathways. For instance, IL-6 has been observed to amplify toll-like receptor (TLR)-mediated cytokine production, suggesting its role in exacerbating inflammatory responses in cancer pain contexts [91–94]. IL-1β is another key player in the inflammatory milieu associated with cancer pain. It is known to promote pain by sensitizing nociceptive neurons. The release of IL-1β can lead to the activation of various signaling pathways that contribute to the sensation of pain, making it a target for therapeutic intervention in managing cancer pain [92–94]. TNF-α is also implicated in the development of cancer pain. It participates in recruiting immune cells to the tumor microenvironment. It participates in recruiting immune cells to the tumor microenvironment and could induce pain by acting on peripheral nociceptors. The modulation of TNF-α levels has been shown to correlate with pain intensity in cancer patients, highlighting its potential as a therapeutic target [92–95]. C C motif ligand 2 (CCL2), a chemokine, is known for its role in recruiting monocytes to sites of inflammation. CCL2 could facilitate the infiltration of immune cells into the tumor microenvironment, contributing to the inflammatory response and cancer pain sensation. Elevated CCL2 levels have been linked to enhanced pain perception in cancer patients, indicating its relevance in pain mechanisms [96, 97]. Prostaglandin E2 (PGE2), a lipid mediator, is another critical component in the pain pathway. It is produced in response to inflammatory stimuli and is known to sensitize nociceptive neurons, thereby enhancing pain perception. PGE2 acts through its receptors to modulate pain signaling pathways, making it a significant mediator in cancer pain [97, 98]. Granulocyte macrophage-colony stimulating factor (GM-CSF) is recognized for its role in the differentiation and activation of myeloid cells, which can influence the inflammatory response in cancer. Its involvement in enhancing the secretion of IL-1β suggests that GM-CSF might be implicated in enhancing pain signaling within the tumor microenvironment [97, 99]. Bradykinin, a peptide that promotes vasodilation and increases vascular permeability, is also implicated in the pain response. It may increase the sensitivity of nociceptors and promote cancer pain development. The interaction of bradykinin with other inflammatory mediators can exacerbate pain, making it an important factor in the overall pain experience in cancer [97, 100, 101].

**Fig. 3:**
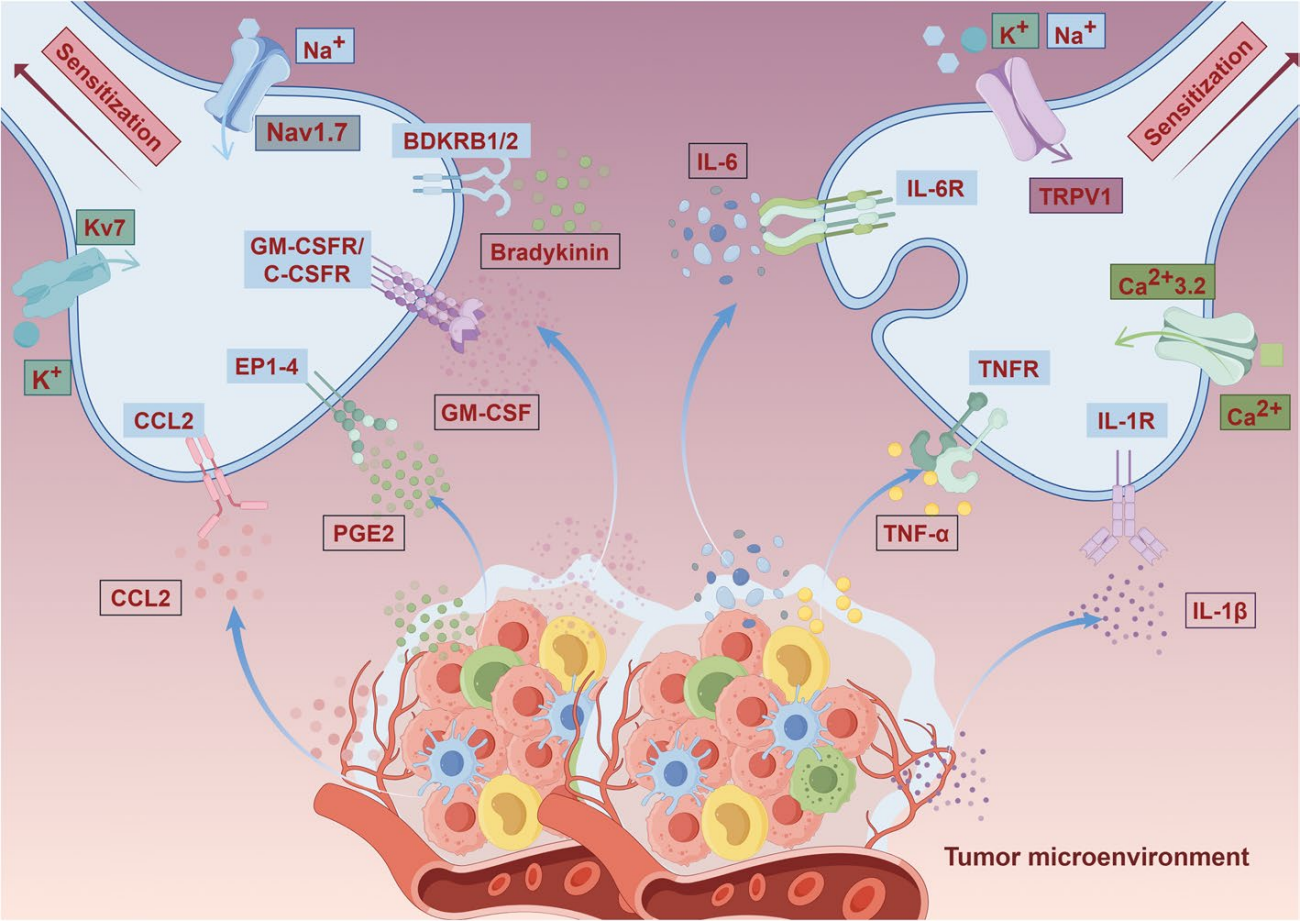
Molecular mechanisms of peripheral sensitization in cancer pain. The tumor microenvironment constitutes a dynamic cellular ecosystem that secretes multiple algogenic mediators that collectively induce peripheral sensitization of tumor-innervating sensory neurons. Pro-nociceptive factors including CCL2, PGE2, GM-CSF, IL-6, TNF-α and IL-1β bind to their cognate receptors, triggering downstream phosphorylation events that lower neuronal activation thresholds. This results in enhanced signal transduction through downstream pathways. Concurrently, Nav1.7 undergoes phosphorylation-induced activation while Kv7 experiences functional downregulation. These changes synergize with TRPV1 sensitization and Ca^2^⁺3.2 activation to generate ectopic action potentials. The resultant neuronal hyperexcitability manifests clinically as spontaneous pain and mechanical hyperalgesia

In conclusion, the interplay between these inflammatory mediators—IL-6, IL-1β, TNF-α, CCL2, PGE2, GM-CSF, and bradykinin—highlights their collective role in the mechanisms underlying cancer pain (Fig. 3). Targeting these mediators may offer new avenues for therapeutic interventions aimed at alleviating pain in cancer patients. Further studies are required to clarify the exact manner in which these mediators interact and contribute to the pain experience in cancer.

#### Ion channels

Voltage-gated ion channels are key to neuronal excitability and nerve signal transmission. Typically, ion flux regulation through these transmembrane proteins is precisely coupled to action potential propagation through dynamic sensitivity to transmembrane voltage fluctuations in excitable cellular systems, allowing ions such as sodium (Na**⁺**) and calcium (Ca^2^**⁺**) to enter and exit the cell, triggering action potentials and transmitting nerve signals [102–104]. This mechanism is crucial for the effective operation of the nervous system. However, the function of voltage-gated ion channels is often disrupted, leading to excessive activation of the nervous system and, consequently, persistent cancer pain (Fig. 3). Studies have shown that blocking Na^+^ channels, particularly Nav1.7, effectively reduces paclitaxel-induced hyperexcitability of Dorsal root ganglion (DRG) neurons and alleviates chemotherapy-induced pain. Therefore, Nav1.7 has emerged as a potential target for cancer pain therapy [105]. Beyond sodium channels, potassium ion channels also are key contributors to cancer pain. In particular, abnormal expression of the Kv7 channel subtype has been linked to neuropathic pain. Research has demonstrated that chemotherapy drugs such as oxaliplatin and paclitaxel downregulate Kv7 channel expression in cortical neurons and DRG neurons, leading to depolarization and excessive excitation of these neurons, thereby promoting pain. Notably, the Kv7 channel activator retigabine has been shown to effectively alleviate pain in a paclitaxel-induced neuropathy mouse model, suggesting that Kv7 channels serve as a potential therapeutic targe for cancer-related pain [106]. Calcium channels also play an important role in cancer pain. Chemotherapy drugs like paclitaxel can alter the function of calcium channels, particularly the T-type calcium channel Ca^2**+**^3.2, leading to persistent neuronal excitation. The expression of Ca^2**+**^3.2 is significantly increased in animal models, which is closely associated with the onset of pain. Inhibiting this channel has been shown to effectively reverse hypersensitivity, highlighting the critical role of T-type calcium channels in cancer pain [107–109].

Additionally, transient receptor potential vanilloid (TRPV) channels, particularly TRPV1, have gained considerable attention in the context of cancer pain, especially CIPN. It has been thermal hypersensitivity, pain sensitization and aberrant mechanical responses in sensory neural pathways induced by chemotherapy drugs such as cisplatin, oxaliplatin, bortezomib, and paclitaxel [110–112]. The TRPV1 channels is a major driver of cancer-related pain. Clinical trials involving oxaliplatin-induced peripheral neuropathy have shown that modulating the function of TRPA1 channels can prevent the onset of pain symptoms, further supporting the critical role of TRP channels in cancer pain [113, 114].

### Central sensitization

#### Neurotransmitter dysregulation

**Fig. 4:**
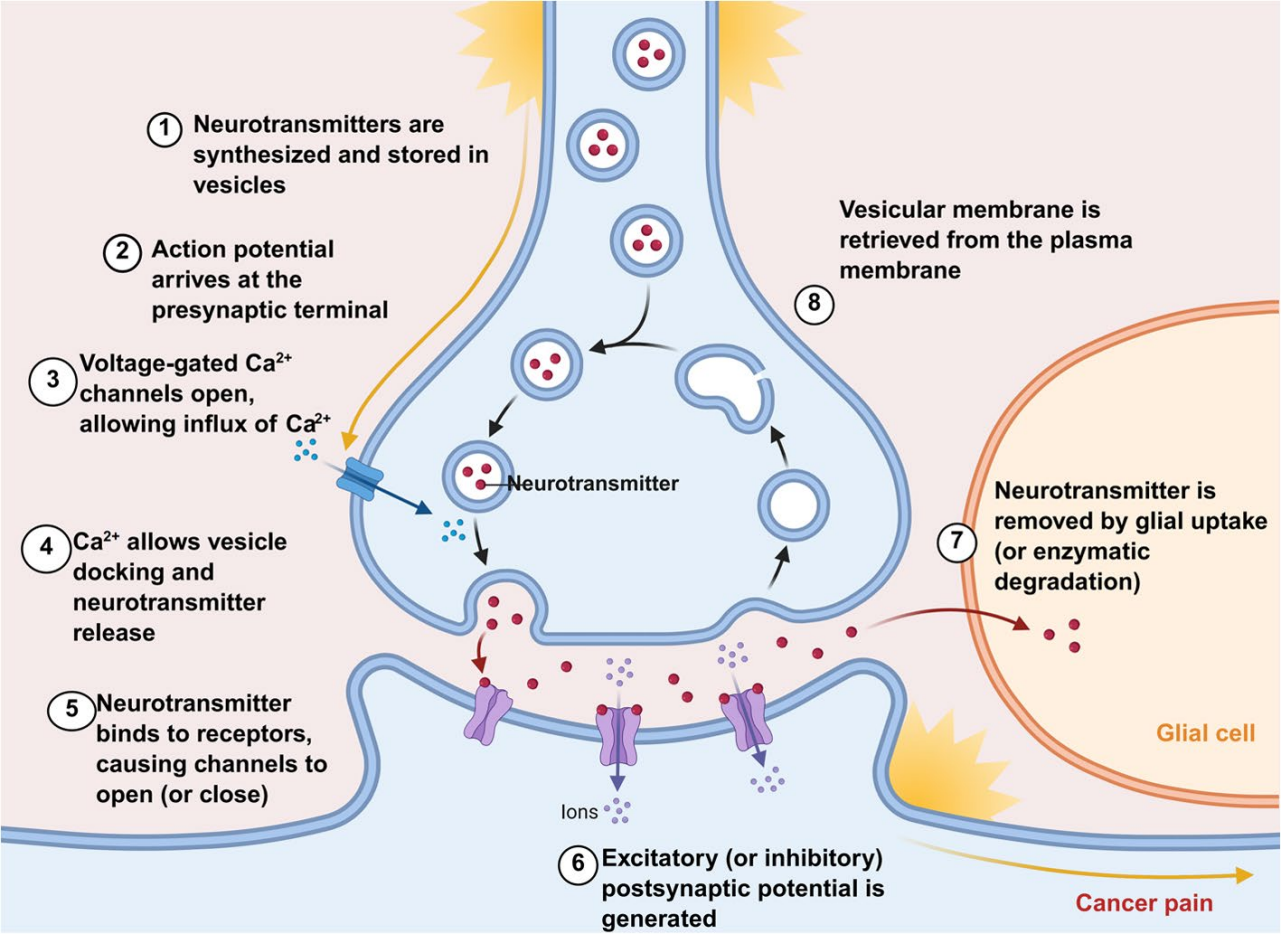
Synaptic transmission mechanisms underlying central sensitization in cancer pain. The development of central sensitization in cancer pain is largely dependent on synaptic communication within the CNS. This process, which includes synaptic plasticity and neurotransmitter release, can lead to heightened cancer pain perception when there is prolonged or excessive activation. In cancer pain, these mechanisms are often dysregulated, resulting in abnormal presynaptic glutamate release and postsynaptic receptor hypersensitivity. Consequently, sustained neuronal hyperexcitability occurs, ultimately leading to chronic cancer pain states. Created in BioRender (2025) https://BioRender.com/7ko8rh8

The importance of neurotransmitters in cancer pain is a complex research field that has attracted much attention in recent years. Neurotransmitters are crucial in modulating pain pathways, and their involvement in cancer-related pain mechanisms is particularly noteworthy. Various neurotransmitters, including serotonin, dopamine, and glutamate, have been implicated in the modulation of pain perception and the development of pain syndromes associated with cancer (Fig. 4).

One of the key neurotransmitters involved in cancer pain is glutamate, which acts as the primary excitatory neurotransmitter in the central nervous system (CNS). Tts dysregulation can lead to heightened pain sensitivity, a phenomenon often observed in cancer patients. Studies have shown that increased glutamate levels in the tumor microenvironment can contribute to the sensitization of pain pathways, leading to chronic pain conditions associated with cancer [115–117]. Additionally, the endogenous opioid system, which includes neurotransmitters such as beta-endorphins and enkephalins, is critical for modulating pain. The network between opioids and their receptors can significantly influence the perception of pain in cancer patients. Research indicates that the administration of opioids can lead to alterations in neurotransmitter release, which may help alleviate cancer pain. However, the long-term use of opioids is often associated with tolerance and dependence, complicating pain management strategies [118–121]. Moreover, the serotonergic system has been identified as a potential target for pain management in cancer patients. Moreover, pharmacological modulation of serotonergic system may offer novel therapeutic strategies for controlling malignancy-associated pain. Serotonin is known to modulate pain pathways and can influence the efficacy of analgesics. Some studies suggested that enhancing serotonergic transmission may improve pain relief in patients suffering from cancer-related pain [122, 123]. The interplay between serotonin and other neurotransmitters, such as norepinephrine, further complicates the understanding of cancer pain modulation. The tumor microenvironment also influences neurotransmitter dynamics, with evidence suggesting that cancer cells could alter the release and uptake of neurotransmitters, thereby affecting pain signaling pathways. For instance, neurotransmitters like adenosine triphosphate (ATP) and norepinephrine could modulate immune responses, which can subsequently impact pain perception and progression [124].

#### Glial cell activation

Microglia, as inherent immune cells of the CNS and part of the mononuclear phagocyte system, sense environmental changes and dynamically polarize into two functionally distinct M1 and M2 phenotypes [125, 126]. Astrocytes, the most abundant cells in the CNS, with a structure closely linked to synaptic structures, facilitate neuronal circuit formation and maintain synaptic integrity [127, 128]. The evidence highlights the crucial role of microglia and astrocytes in cancer pain. They contribute to and exacerbate central sensitization through mutual activation and inflammatory mediator release. It involves activating multiple signaling pathways and complex interactions between glial and neuron cells. The tumor microenvironment releases numerous molecular signals that directly activate glial cells. In bone cancer pain (BCP) models, tumor-mediated bone destruction produces lactate, PGE2, extracellular ATP, and high-mobility group box 1 (HMGB1). They stimulate microglial purinergic receptors (purinergic receptor p2x ligand-gated ion channel 4/ purinergic receptor p2x ligand-gated ion channel 7, P2X4/P2X7) and toll-like receptor 4 (TLR4), inducing them to the pro-inflammatory M1 phenotype [129–131]. Simultaneously, astrocyte activation occurs through neuronal chemokines (C-X3-C motif chemokine ligand 1, CX3CL1) or IL-1β, TNF-α, characterized by increased glial fibrillary acidic protein (GFAP) expression and cellular hypertrophy [132–134]. Importantly, glial activation shows spatiotemporal dynamics: microglia respond rapidly in acute pain phases, while astrocyte activation correlates with chronic pain maintenance.

Activated glia enhance nociception by releasing inflammatory mediators that either directly increase neuronal excitability or structurally modify pain transmission pathways. In BCP models, spinal microglia upregulate nuclear factor of activated T cells 2 (NFATC2), triggering p38 mitogen-activated protein kinase/mitogen-activated protein kinase (p38/MAPK) signaling that phosphorylates proteins and releases interleukin-18 (IL-18). It enhances neuronal N-methyl-D-aspartic acid receptor (NMDA) receptor activation, directly contributing to cancer-related hyperalgesia [135]. Studies in BCP models revealed that specific annexin A3 (ANXA3) knockdown in microglia significantly inhibits the hypoxia inducible factor-1α/vascular endothelial growth factor (HIF-1α/VEGF) signaling pathway, which reduced p-protein kinase C (PKC) levels in spinal cord neurons, leading to marked attenuation of cancer progression-related nociceptive responses [136]. Microglia-derived proinflammatory cytokines exert pleiotropic effects on neuronal activity, thereby potentiating nociceptive signal transduction and facilitating central sensitization in pain pathways [133, 137, 138]. In BCP models, interleukin-17A (IL-17A) released by activated astrocytes in the spinal dorsal horn mediates central sensitization through activation of neuronal interleukin-17 receptor A/pCalcium Calmodulin Dependent Protein Kinase IIα (IL-17RA/pCaMKIIα signaling axis, thereby modulating nociceptive transmission [139]. C-X-C motif chemokine ligand 1/12 (CXCL1/CXCL12) secreted by astrocytes specifically bind to their cognate receptors C-X-C motif chemokine receptor 2/4 (CXCR2/CXCR4) on nociceptive neurons, respectively, which downregulates neuronal excitability through membrane hyperpolarization, consequently potentiating central sensitization of nociceptive transmission [140, 141].

**Fig. 5:**
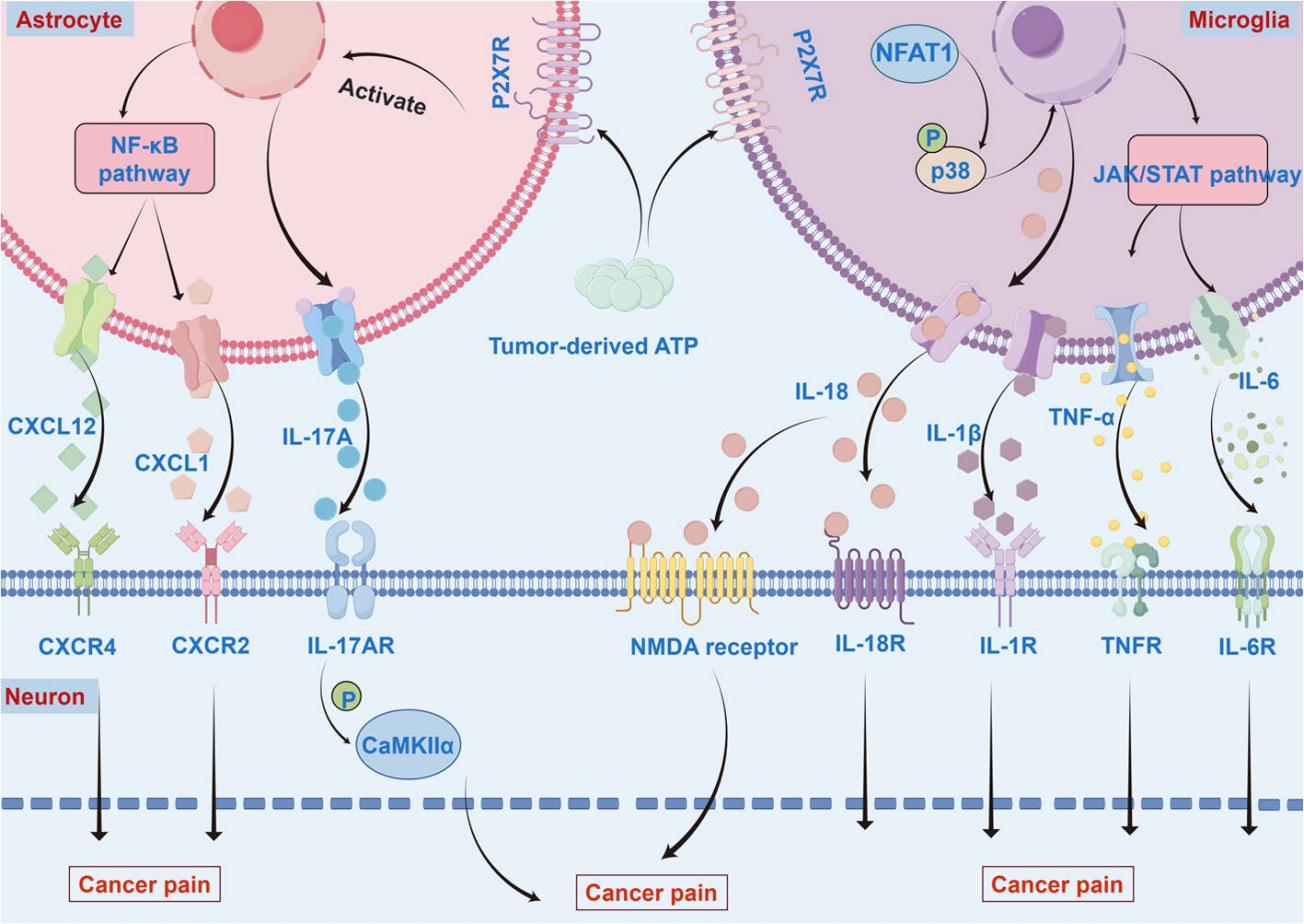
Glia-neuron interplay drives central sensitization in cancer pain. Astrocytic reactivity mediated through P2X7 receptors initiates CXCL12 and CXCL1 chemotaxis and IL-17A-mediated inflammatory responses. Microglial polarization via specific receptors triggers IL-1β, IL-18 and IL-6 release. Chemokine-inflammatory networks engage neuronal membrane receptors, enhancing pain signaling. Concurrent NF-κB (transcriptional activation) and JAK2-STAT3 (phosphorylation cascade) pathways synergistically upregulate pro-nociceptive gene clusters. The interplay among astrocytes, microglia, and neurons creates a microenvironment sustaining inflammatory and cancer pain mechanisms

In the pathogenesis of pain, glial activation within the CNS emerges as a central pathological determinant. Particularly: The janus kinase/ signal transducer and activator of transcription (JAK/STAT) cascade drives proinflammatory cytokine production via signal transducer and activator of transcription 3 (STAT3) phosphorylation [142–144]; Nuclear factor kappa-light-chain-enhancer of activated B cells (NF-κB) signaling orchestrates neuroinflammation through transcriptional control of inflammatory mediators [145, 146]; p38/MAPK pathway modulates neuronal hypersensitization [147–149]; Phosphatidylinositol 3-kinase/Ak strain transforming (PI3K/Akt) signaling axis exacerbates pain progression through maladaptive synaptic plasticity [150, 151]. Mechanistically, neuro-glial crosstalk networks amplify nociceptive processing through coordinated cellular interactions (Fig. 5).

### Cellular stress and dysfunction

#### Endoplasmic reticulum stress

The role of endoplasmic reticulum (ER) stress in cancer pain mechanisms has garnered increasing attention. ER stress is a cellular condition that arises when there is an accumulation of misfolded or unfolded proteins in the ER, triggering a protective response known as the unfolded protein response (UPR). This response can have dual outcomes: it can promote cell survival under stress conditions or lead to apoptosis if the stress is unresolved [152–154].

Recent studies have highlighted that ER stress plays a critical role in the development of opioid-induced hyperalgesia (OIH), a paradoxical increase in pain sensitivity following opioid treatment. For instance, chronic morphine administration has been shown to upregulate ER stress markers like glucose regulatory protein 78 (GRP78) in spinal neurons, indicating that opioids can exacerbate ER stress, which in turn enhances pain signaling pathways. The activation of unfolded protein response (UPR) signaling pathways, particularly the inositol-requiring enzyme 1α/x-box binding protein 1 (IRE1α/XBP1) and protein kinase r-like endoplasmic reticulum kinase/eukaryotic translation initiation factor 2 subunit α (PERK/eIF2α) pathways, has been implicated in the modulation of pain responses, suggesting that targeting these pathways could provide new therapeutic strategies for managing cancer pain [155, 156]. Moreover, the interplay between ER stress and inflammation in the tumor microenvironment is another critical aspect of cancer pain. Tumor cells can manipulate ER stress responses to evade immune detection and promote a pro-inflammatory environment, which can contribute to pain. For example, ER stress has been shown to influence the secretion of inflammatory cytokines, which can sensitize nociceptive pathways and exacerbate pain [157, 158]. It suggests that interventions aimed at modulating ER stress responses could potentially alleviate cancer pain by restoring immune function and reducing inflammation.

#### Mitochondrial dysfunction

Current research delineates compromised mitochondrial function as an essential mediator in the progression of oncological pain syndromes. Mitochondria are not only the powerhouses of cells, generating ATP through oxidative phosphorylation, but they also play vital roles in regulating apoptosis and reactive oxygen species production. The mitochondrial dysfunction could lead to increased oxidative stress, which has been implicated in the sensitization of nociceptive pathways in tumor. Studies have shown that chemotherapeutic agents, such as paclitaxel and oxaliplatin, induce mitochondrial damage, leading to peripheral neuropathy characterized by pain [159, 160]. This is further supported by findings that mitochondrial poisons exacerbate pain symptoms in models of chemotherapy-induced neuropathy, indicating a direct link between mitochondrial health and pain perception [161, 162]. What’s more, alterations in mitochondrial function can influence the activation of pro-inflammatory signals, further contributing to the patients’ pain experience [163, 164].

#### Microtubule damage

Microtubules, composed primarily of α-tubulin and β-tubulin, are pivotal for maintaining cellular functions through their dynamic equilibrium [165, 166]. Within neurons, the integrity of microtubules is crucial for the maintenance of axonal transport and synaptic functions. When microtubules are compromised, the transport and release of neurotransmitters can be disrupted, leading to aberrant neural signaling and impaired pain signal transmission [167, 168]. Moreover, microtubule damage may trigger intracellular stress pathways, which are implicated in cancer pain sensitization [169, 170]. Moreover, the growth and invasion of tumor cells can directly injure nerve fibers and disrupt microtubule structures [171]. This microtubule damage not only impairs neuronal function but may also exacerbate pain response by releasing inflammatory mediators and nerve growth factors.

### Signaling pathways associated with cancer pain

#### PI3K/Akt/mTOR signaling pathway

One significant aspect of the pathway is its contribution to the development of cancer pain through the modulation of inflammatory responses and neuronal sensitization. In particular, the activation of this pathway has been linked to the production of pro-inflammatory cytokines, which could enhance pain signaling in the nervous system. For instance, the dysregulation of the pathway has been observed in various cancers, leading to increased levels of inflammatory mediators that sensitize nociceptive neurons, thereby exacerbating pain perception in cancer patients [172, 173]. Moreover, the pathway is implicated in the survival and proliferation of cancer cells, which could indirectly contribute to pain by promoting tumor growth and metastasis. Tumors could exert pressure on surrounding tissues and nerves, leading to pain. It’s role in tumorigenesis highlights the potential of PI3K/Akt/mTOR inhibitors not only in treating cancer but also in alleviating associated pain [174, 175]. What’s more, recent clinical trials have explored the efficacy of combining PI3K/Akt/mTOR inhibitors with other therapeutic agents to improve pain management in cancer patients. These combination therapies aim to enhance the overall therapeutic effect while minimizing side effects, thus providing a more comprehensive approach to cancer treatment [172].

#### TGF-β signaling pathway

Recent studies have highlighted that transforming growth factor β (TGF-β) signaling is involved in the development of cancer-related pain through its effects on the tumor microenvironment. For instance, it could promote the recruitment of immune cells and the formation of a fibrotic stroma, which can lead to increased pressure on surrounding nerves and tissues, thereby exacerbating pain sensations. Additionally, TGF-β has been shown to modulate the release of pain-related molecules, including nerve growth factor (NGF) and other neurotrophic factors, which can sensitize nociceptive pathways and contribute to the development of hyperalgesia in cancer patients [176, 177]. In addition to its role in pain modulation, TGF-β signaling is also critical in the context of cancer metastasis. This process supports the epithelial-mesenchymal transition (EMT), enabling cancer cells to invade nearby tissues and metastasize to distant locations. This invasive behavior not only contributes to the progression of cancer but also to the development of pain associated with metastatic lesions. Understanding the mechanisms by which TGF-β signaling promotes EMT and metastasis can provide insights into potential interventions that could alleviate pain by targeting these processes [178, 179].

#### Notch signaling pathway

Notch signaling has been shown to influence the tumor microenvironment and the interactions between cancer cells and surrounding tissues in cancer pain. For instance, the activation of Notch in the anterior cingulate cortex has been implicated in the development of neuropathic pain, suggesting that it may play a role in the central processing of pain signals in cancer patients [180]. Furthermore, it can modulate the immune response within the tumor microenvironment, affecting the recruitment and activation of immune cells that contribute to pain sensation [181]. Moreover, the crosstalk between Notch signaling and other pathways, such as the TGF-β pathway, has been identified as a crucial mechanism in cancer pain modulation. Notch signaling can inhibit TGFβ activity, which is known to be involved in pain pathways, thereby influencing the overall pain response in cancer [182]. This interplay highlights the complexity of Notch signaling in cancer biology and its potential as a therapeutic target for alleviating cancer-related pain.

### Epigenetic regulation

Cancer pain is typically caused by tumor compression, invasion of surrounding tissues, or interactions between cancer cells and the immune system, leading to chronic hypersensitivity to pain. The epigenetic mechanisms may be involved in the regulation of gene expression, immune responses, and the sensitivity of the nervous system in cancer pain.

#### DNA methylation

It has been shown that DNA methylation alterations can affect the expression of genes involved in nociception, the sensory perception of pain. Chronic pain syndromes often exhibit distinct DNA methylation profiles that correlate with hypersensitivity to painful stimuli and neuronal hyperexcitability. The regulation of pro- and anti-nociceptive genes through DNA methylation may thus represent a critical mechanism by which cancer pain is exacerbated [183, 184]. In addition, the involvement of DNA methyltransferases (DNMTs) in the regulation of pain-related genes has been documented. DNMTs are responsible for adding methyl groups to DNA, and their dysregulation can lead to altered pain sensitivity. For example, the inhibition of DNMT activity has been shown to reverse aberrant methylation patterns, restoring the expression of genes that may mitigate pain responses. It suggests that pharmacological modulation of DNA methylation could be a viable approach for managing cancer pain [185].

#### Histone modifications

The modification of histones by acetylation and methylation has been shown to affect the transcriptional activity of genes associated with pain pathways. In a mouse model of chronic postsurgical pain, it was observed that the expression levels of Sprr1a and Anxa10, which are key modulators of neuropathic pain, were significantly increased in the spinal cord following electrocautery. This increase was associated with a decrease in tri-methylated histone H3 at Lys27 and an increase in acetylated histone H3 at Lys27 at their promoter regions, suggesting that histone modifications directly influence the expression of these pain-related genes [186]. Moreover, the role of histone deacetylases (HDACs) and their inhibitors has been explored in the context of cancer pain. HDACs are known to regulate histone acetylation, which in turn affects gene expression related to pain signaling pathways. Inhibition of HDACs has been shown to alter the expression of genes involved in pain mechanisms, thereby providing a potential therapeutic avenue for managing cancer-related pain [187]. Additionally, the epigenetic landscape in cancer is often altered, leading to dysregulation of pain-related gene expression. For example, the aberrant expression of histone methyltransferases, such as enhancer of zeste 2 polycomb repressive complex 2 subunit (EZH2), has been implicated in the progression of breast and colorectal cancers. These enzymes modify histones in a way that could enhance the transcription of oncogenes while silencing tumor suppressor genes, which may indirectly contribute to the development of pain associated with tumor growth and metastasis [188, 189].

#### MicroRNAs

MicroRNAs (miRNAs) have emerged as critical regulators in various biological processes, including the pathogenesis of cancer and its associated pain. The miR-34c-5p has been identified as a pronociceptive miRNA that targets calcium channels, thereby influencing pain sensitivity in cancer models [190]. This suggests that miRNAs can directly impact nociceptive signaling, contributing to the heightened pain experienced by cancer patients. Moreover, the dysregulation of specific miRNAs has been correlated with the pain development. For example, a study demonstrated that miR-330 is significantly upregulated in the spinal dorsal horn of mice with pancreatic cancer pain, where it modulates the expression of gamma-aminobutyric acid b receptor subunit 2 (GABABR2), a receptor involved in pain signaling [191]. This indicates a potential mechanism by which cancer can induce pain through miRNA-mediated pathways, emphasizing the need for further exploration of miRNA functions in pain modulation. Furthermore, the expression of miRNAs such as miR-101 has been shown to attenuate neuropathic pain through the downregulation of mTOR, a key player in pain signaling pathways [192]. This suggests that targeting specific miRNAs could provide a novel therapeutic strategy for managing cancer-related pain, potentially enhancing the patients’ life.

## Cancer pain assessment

Cancer pain assessment plays a vital role in the clinical treatment of cancer patients. It helps not only to understand the severity of a patient’s pain comprehensively but also to provide a theoretical basis for developing personalized pain management strategies. Cancer pain is complex and diverse, making its assessment particularly challenging. Pain assessment must, therefore, adhere to scientific principles such as comprehensive, dynamic and quantitative. A holistic approach is required, taking into account various factors to assess the type, intensity, and nature of the pain. Appropriate tools should be used during this process to quantify and effectively describe the pain [193–195].

### Principles of cancer pain assessment

Comprehensive assessment of cancer pain characteristics: The first step in the comprehensive assessment of pain characteristics is to conduct a thorough and systematic evaluation of the cancer patient’s pain and related conditions. This involves understanding the etiology, type, nature, duration, and frequency of the cancer pain [196, 197]. Additionally, the patient’s overall bodily functions should be evaluated, with a focus on vital organs such as the liver, kidneys, and heart.

Multidimensional assessment: The assessment of cancer pain should encompass multiple dimensions, including physical, psychological, social, and emotional aspects, in order to fully understand the sources of the patient’s suffering. Pain is not just a physical sensation; it may also be associated with emotional issues, including anxiety, depression and insomnia, which could significantly affect the patient’s overall health [198].

Patient participation: The patient is the primary source of cancer pain perception, and their self-assessment often yields more valuable information than a physician's evaluation [199]. Therefore, the assessment process should incorporate the patient's self-reports in full.

Continuous assessment and adjustment: Cancer pain is typically persistent and fluctuating, with clinical characteristics that can evolve over time. Pain assessment should be an ongoing process, regularly updated using standardized tools [200, 201]. The frequency of assessments can be adjusted according to the patient’s evolving condition to ensure effective pain management.

Interdisciplinary collaboration: The assessment and management of cancer pain require collaboration among a multidisciplinary team, including professionals from oncology, anesthesiology, pain management, psychology, and pharmacy [202, 203]. This team approach helps assess the patient’s pain from various perspectives and provides a comprehensive treatment plan.

### Cancer pain assessment content

Assessing the type of pain in is a crucial first step in treatment, as different pain types necessitate distinct management strategies. Cancer pain is usually classified into 2 main types: nociceptive and neuropathic pain [204]. Nociceptive pain is often described as dull, aching, throbbing, or sharp [204, 205]. Neuropathic pain, on the other hand, is marked by sensations such as burning, electric shock-like pain, or tingling [204, 206]. It is critical to emphasize that most cancer patients experience a combination of nociceptive and neuropathic pain, rather than just one type. Pain intensity is one of the most commonly used indicators in pain assessment. Pain intensity is typically assessed using unidimensional tools, with common parameters such as current pain intensity, average pain over the past 24 h, worst pain, least pain, and intensity under different conditions (at rest vs during activity) [207]. Patients typically report their pain intensity through self-assessment, which is commonly graded using standardized scoring systems, most often the 0–10 Numerical Rating Scale (NRS) [208]. In this system, 0 indicates no pain, and 10 represents the most severe pain, with patients rating their pain based on subjective experience. Visual Analog Scale (VAS) constitutes a principal metric in which subjects graphically map subjective pain magnitude between defined poles of “pain-free” and “worst conceivable pain” [209]. Another important aspect of cancer pain assessment is evaluating the pain's duration and frequency, which include intermittent and persistent pain. Intermittent pain refers to pain that occurs at specific times or during certain activities, typically lasting for shorter durations with variable intensity [207]. Persistent pain, on the other hand, refers to continuous pain with little to no relief in both resting and active states, and its intensity remains relatively stable [194]. Cancer pain assessment goes beyond evaluating the type, intensity, and duration of pain; it also involves considering other factors [207]. The assessment should include evaluating the skin and mucous membranes for any pathological changes, such as redness, rashes, ulcers, or lumps. It is also important to assess whether the patient has any comorbidities, such as gastrointestinal ulcers, bleeding disorders, or anticoagulant use. Laboratory tests (such as blood counts and liver/kidney function tests) and imaging studies (computed tomography/magnetic resonance imaging, CT/MRI) should also be part of the assessment, as they help determine cancer progression and whether metastasis or other lesions are present, providing insights into the source and nature of pain.

In addition to physiological factors, pain assessment must also consider the patient’s psychological state and social environment. Psychological factors often exacerbate pain sensitivity and the perception of suffering. Social support systems are also crucial in the assessment. Family support, social interactions, and the living environment can significantly influence pain perception and coping abilities [68, 210–212].Therefore, cancer pain assessment should be a multidimensional and comprehensive process that incorporates physiological, psychological, and social factors for holistic pain management. By thoroughly assessing each dimension, a more precise and personalized treatment plan can be developed, not only alleviating.

### Tools for cancer pain assessment

Common tools for unidimensional pain intensity assessment include the NRS and the VAS. The NRS requires patients to perform quantitative self-assessment along a 0–10 integer-based continuum, where terminal anchors demarcate pain-free status (0) and incapacitating symptom severity (10) [208]. For patients with communication challenges, such as children, the elderly, or those with impaired consciousness, the Faces Pain Scale (FPS) is recommended [213]. It evaluates pain by observing facial expressions, which is especially useful for those who cannot reliably communicate their pain intensity. It is highly practical and effective in clinical settings. The Brief Pain Inventory (BPI) is an essential tool for multidimensional cancer pain assessment [214]. The BPI not only assesses pain intensity but also evaluates its impact on daily life, including sleep, activities, and mood. For critically ill patients unable to self-report pain, the Multidimensional Objective Pain Assessment Tool (MOPAT) was developed [215]. This tool combines physiological signals and clinical observations to assess changes in the patient before and after pain interventions, making it particularly useful for non-verbal patients, such as those in critical care or with impaired consciousness. In addition to assessing pain intensity, the psychological distress of cancer patients must also be addressed. Emotional issues often accompany cancer pain and can intensify pain perception. Thus, evaluating the patient's psychological state is critical. Commonly used psychological assessment tools include the Distress Thermometer (DT), Patient Health Questionnaire-9 (PHQ-9) and Generalized Anxiety Disorder-7 (GAD-7) [216–218]. Furthermore, assessing breakthrough cancer pain requires specialized tools. Breakthrough pain refers to a sudden worsening of pain in patients who are already undergoing pain management. The Breakthrough Pain Assessment Tool (BAT) has been translated and validated, showing strong reliability and validity [219]. These tools (Table 2) help clinicians identify and adjust treatment strategies promptly to better address this clinical challenge.

**Table 2:** The basic characteristics of pain assessment tools

Tool	Type	Assessment content	Patient population	Ref
NRS	Single-dimensional pain intensity assessment	Pain intensity (on a scale of 0–10, 0 being painless and 10 being the most intense pain)	Cancer patient could clearly express pain intensity	[208]
VAS	Single-dimensional pain intensity assessment	Pain intensity is indicated by a mark on the scale	Cancer patients could understand and use visual scales	[209]
FPS	Single-dimensional pain intensity assessment	Pain intensity is assessed by looking at facial expressions	Children and elderly, cancer patients with confusion or difficulty communicating	[213]
BPI	Multi-dimensional pain intensity assessment	Pain intensity, interference with daily activities (sleep, activity, mood)	All cancer patients, impact of pain on quality of life	[214]
MOPAT	Multi-dimensional pain intensity assessment	Changes, physiological signals and clinical observation of pain before and after intervention	Seriously ill, impaired consciousness, and unable to self-report pain cancer patients	[215]
DT	Psychological assessment	Assess the patient's emotional distress (anxiety, depression, etc.)	All cancer patients	[216]
PHQ-9	Psychological assessment	Assess depressive symptoms and their severity	Cancer patients, especially those with depressive symptoms	[217]
GAD-7	Psychological assessment	Generalized anxiety symptoms and their severity are assessed	Cancer patients, especially those with anxiety	[218]
BAT	Specific pain types assessment	Assess the characteristics and severity of cancer breakthrough pain	Cancer patients, especially those with breakthrough pain	[219]

*NRS* Numerical Rating Scale, *VAS* Visual Analogue Scale, *FPS* Faces Pain Scale, *BPI* Brief Pain Inventory, *MOPAT* Multidimensional Objective Pain Assessment Tool, *DT* Distress Thermometer, *PHQ-9* Patient Health Questionnaire-9, *GAD-7* Generalized Anxiety Disorder-7, *BAT* Breakthrough Pain Assessment Tool

## Pharmacological management of cancer pain

**Fig. 6:**
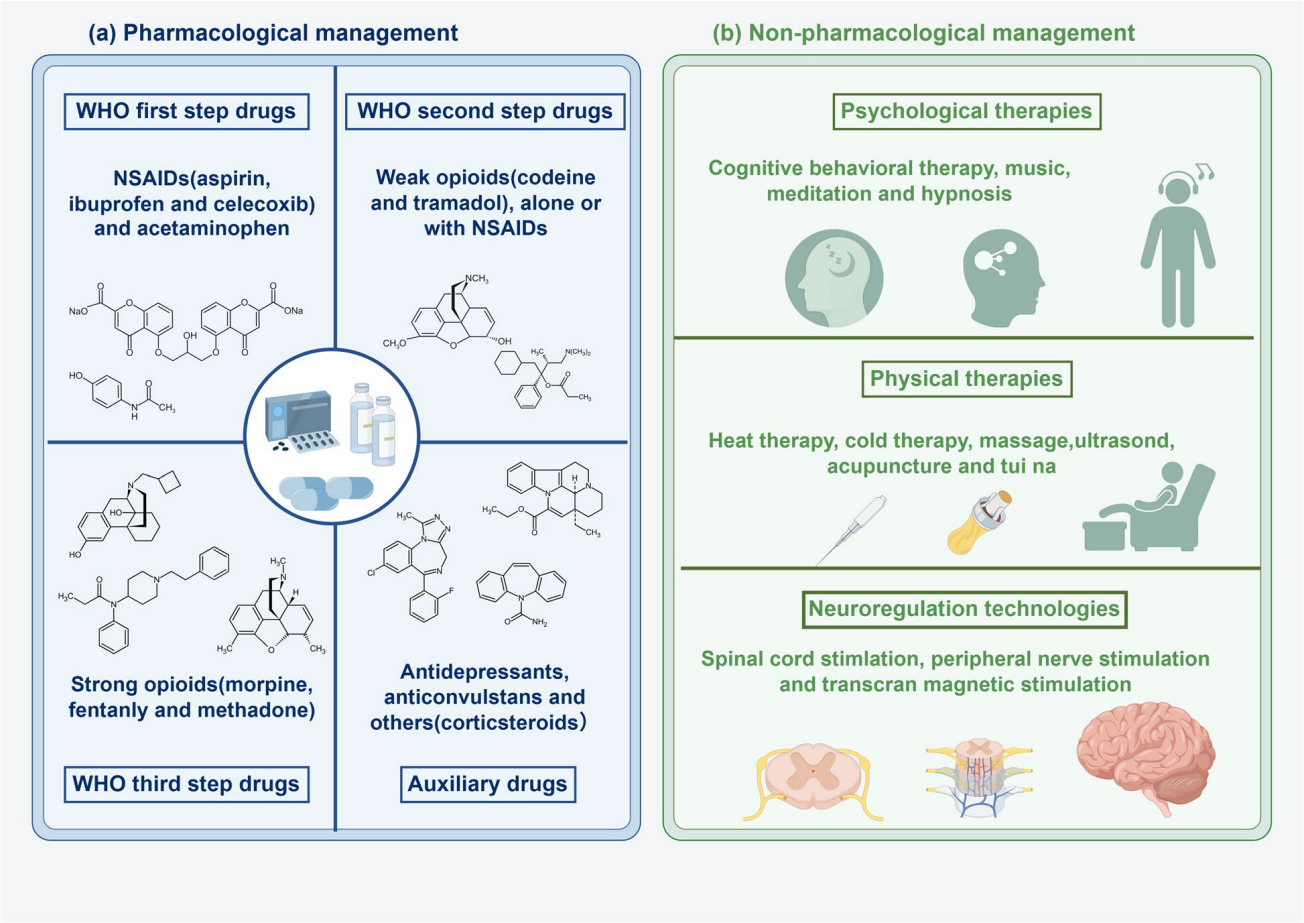
Cancer pain management strategies. **a** Pharmacological management follows the WHO analgesic ladder framework. First step: NSAIDs and acetaminophen for mild-moderate nociceptive pain; Second step: weak opioids combined with the first step’s drugs for moderate cancer pain; Third step: Strong opioids for severe persistent cancer pain. Adjuvant therapy: antidepressants, anticonvulsants and corticosteroids to potentiate analgesia and address neuropathic components. **b** Non-pharmacological management includes psychological interventions (CBT, mindfulness meditation, and music therapy to modulate pain perception and improve coping mechanisms), physical modalities (thermotherapy, therapeutic massage, medical acupuncture, and ultrasound therapy for peripheral pain modulation) and neuromodulation techniques (SCS, PNS and TMS for central nervous system pathway regulation)

Pharmacological intervention remains the cornerstone of management. The drug management principles in current guidelines are primarily based on the WHO’s classic “three-step ladder” approach, with ongoing improvements [12–14, 16, 207, 220]. It divides cancer pain management into three stages, progressively increasing the intensity and choice of drugs based on the patient’s pain level [221] (Fig. 6). The first step targets mild pain and recommends non-opioid drugs, such as Nonsteroidal anti-inflammatory drugs (NSAIDs) and acetaminophen, as foundational treatments. These medications effectively relieve mild pain and have anti-inflammatory properties, making them the first choice for managing mild cancer pain. The second step addresses moderate pain, typically using weak opioids, such as codeine or tramadol, alone or with NSAIDs to enhance pain relief. The goal at this stage is to manage moderate pain with weak opioids, minimizing side effects and dependency risks associated with stronger opioids. The third step addresses severe pain, typically managed with strong opioids, such as morphine, fentanyl, or methadone. These drugs provide powerful pain relief and are essential for managing pain in advanced patients, significantly improving their life. This staged approach enables a tailored, stepwise escalation of treatment, ensuring effective pain relief while minimizing side effects.

Different drug administration routes have unique characteristics regarding cancer pain relief, drug absorption, release rates, and side effect management. Common methods outlined in current guidelines include oral, intravenous, transdermal, subcutaneous, intrathecal, and parenteral administration [207, 222]. Oral administration is the most common and convenient method, typically suitable for most cancer patients, especially those who can swallow medications [194, 223]. Oral pharmaceutical formulations comprise multiple delivery systems, including solid dosage forms (tablets and capsules) alongside liquid preparations. Because of its convenience, oral administration is commonly used for long-term cancer pain management. However, it may be less effective for patients with acute pain or those who cannot swallow [224, 225]. Intravenous administration, especially the injection of opioids like morphine or fentanyl, is a fast and efficient method commonly used for patients needing rapid relief from severe pain [226]. It is particularly suitable for acute cancer pain or cases where pain control is challenging. Intravenous administration delivers medication directly into the bloodstream, providing rapid analgesic effects, and is commonly used in hospitals or emergency situations [222, 227]. Transdermal administration is a non-invasive method often used for patients needing continuous, long-term pain relief [228, 229]. For example, fentanyl and lidocaine patches are common forms of transdermal delivery [230, 231]. This method gradually releases medication through the skin, making it suitable for patients who need ongoing support but cannot tolerate frequent dosing. It helps reduce fluctuations in drug levels and maintain stable concentrations, ensuring sustained pain relief. Intrathecal and parenteral administration are used for specific types of pain control. Intrathecal injection is a precise and crucial analgesic method for treating refractory cancer pain, particularly effective when traditional oral or intravenous analgesia fails or causes severe adverse effects. It works by directly injecting drugs into the subarachnoid space, utilizing cerebrospinal fluid circulation to target the spinal dorsal horn and CNS receptors, achieving effective pain relief with minimal dosage [232, 233]. Unlike conventional systemic drug delivery, it significantly reduces systemic drug exposure while increasing drug concentration at the spinal level. It demonstrates sustained efficacy in pain alleviation, with therapeutic benefits persisting longitudinally throughout follow-up assessments [234–236]. Parenteral administration delivers medication via routes other than the gastrointestinal tract and is used for patients with gastrointestinal dysfunction, such as through parenteral nutrition [237, 238]. The choice of drug delivery method for cancer pain management is varied, with each route having distinct indications, advantages, and limitations. The most appropriate method should be selected based on the pain level, drug choice, physical condition, and swallowing difficulties.

The traditional on-demand medication approach, where patients take pain-relieving drugs only when experiencing pain, may lead to ineffective pain management. In contrast, scheduled dosing is essential in managing cancer pain. Scheduled dosing ensures continuous and stable medication release, maintaining a constant drug concentration and preventing sharp declines that could trigger pain [239–241]. The efficacy of opioids (extended-release morphine or fentanyl patches), non-opioid analgesics, and other adjunctive medications is closely linked to their concentration in the bloodstream. If drug concentrations are too low, analgesic effects may not be sustained, leading to pain recurrence. Conversely, excessive drug concentrations may bring about side effects including drowsiness, constipation and respiratory depression [242, 243]. Thus, maintaining stable drug levels is crucial for effective cancer pain management. Scheduled dosing ensures stable drug concentrations, helping prevent pain flare-ups and minimizing side effects. Another advantage of scheduled dosing is that it helps patients clearly understand when to take their medication, reducing reliance on pain as a reminder. This not only standardizes medication adherence but also reduces the likelihood of inadequate pain relief due to missed doses. In this model, patients follow their physician’s instructions and take medication on time, ensuring sustained drug effects and avoiding missed doses [244–246]. In practice, scheduled dosing often involves long-acting medications, particularly opioids. For example, oral extended-release morphine and fentanyl patches provide continuous pain relief for up to 24 h. These long-acting medications steadily release the drug over a set period, maintaining effective concentrations and preventing pain exacerbation from declining drug levels [247, 248]. This method ensures patients receive stable, long-lasting analgesic effects throughout treatment. Table 3 provides detailed guidance on various aspects of common drugs use, including principles, prescriptions, dose titration and adverse reactions.

**Table 3:** The common drugs guidelines management

Drug	Principle of use	Prescription	Dose titration	Adverse reactions
Opioids	Suitable for moderate to severe cancer pain	Morphine, oxycodone, fentanyl, tramadol and hydroxymetol	According to the patient’s reaction to the drug and side effects, evaluate every 3–5 days, and gradually adjust the dose	Respiratory depression, constipation, lethargy, nausea, vomiting, drug resistance, dependence, dizziness, excessive sweating, headache
NSAIDs& acetaminophen	Suitable for mild to moderate cancer pain	Ibuprofen, naproxen, diclofenac, tramadol and celecoxib	If there is no adverse reaction, the maximum daily dose should not exceed the recommended limit every 2–3 days according to the efficacy increment	Stomach pain, stomach ulcers, bleeding, impaired kidney function, hypertension, headache, edema, cardiac events (heart failure, heart attack)
Antidepressant	It is mainly used to relieve cancer-related depression, anxiety and neuropathic pain	Selective 5-HT reuptake inhibitors and tricyclic antidepressants	The initial dose should be increased slowly, small at a time, and the patient's response evaluated. The effect is usually assessed after 2–4 weeks and the dose is adjusted if necessary	Dizziness, dry mouth, insomnia, sexual dysfunction, increased anxiety, irregular heart rate, excessive sedation, weight gain
Anticonvulsant	It is mainly used for neuropathic pain in cancer patients	Gabapentin, pregabalin, phenytoin and sodium	Gradually increase the dose according to tolerance. Regular gabapentin increments are 100-300 mg daily	Drowsiness, dizziness, ataxia, memory loss, mood swings, headache. Neurological side effects are especially important in elderly patients

### Opioids

Opioids are a class of potent analgesic drugs that include natural compounds from opium, semi-synthetic derivatives, and fully synthetic analogs [249, 250]. Opioids relieve pain by binding to opioid receptors, including the μ, κ, δ and pain-sensitizing peptide receptors [251–254]. This binding reduces neuronal excitability by lowering intracellular cAMP levels, opening potassium channels, and closing calcium channels, which decreases pain signal transmission and perception [255]. Additionally, opioids can activate the body’s endogenous pain-relieving systems. By stimulating the μ receptor, opioids enhance the pain-relieving effects of endogenous opioid peptides [118, 256].

According to guidelines from organizations like JSPM, WHO and NCCN, the current therapeutic paradigm prioritizes opioid based regimens as the initial intervention strategy for neoplastic pain syndromes exceeding moderate severity thresholds [15–17]. WHO’s analgesic ladder principles dictate that opioid selection algorithms must integrate therapeutic targets with individualized risk–benefit assessments. Short-acting opioids are typically used for rapid titration of pain relief and breakthrough pain, while long-acting formulations are preferred for maintenance therapy, providing stable analgesia over time. However, opioid rotation may be necessary to manage uncontrolled pain or opioid-induced neurotoxicity when side effects occur. Approximately 30% of patients undergo opioid rotation in clinical practice [257]. This involves transitioning from a pure opioid agonist to a mixed agonist–antagonist (pentazocine, buprenorphine, or nalbuphine), a process that requires careful clinical consideration. Although mixed agonist-antagonists may provide analgesia for some patients, they can also induce withdrawal symptoms in opioid-dependent individuals [258, 259]. In some cases, buprenorphine, a mixed agonist–antagonist, has been approved for treating chronic pain in cancer patients [260]. However, buprenorphine may cause withdrawal symptoms, resulting in patient discomfort. More importantly, buprenorphine exhibits a “ceiling effect,” meaning its analgesic effect does not significantly increase with higher doses, potentially limiting its clinical use [261]. In addition to oral formulations, transdermal patches of fentanyl and buprenorphine are ideal for patients who cannot take oral medications or have absorption issues. These patches slowly release medication through the skin, providing continuous pain relief and eliminating the need for oral administration [262, 263]. In recent years, fixed-ratio combinations of short-acting or long-acting oxycodone-naloxone have been developed. These combinations use naloxone to locally antagonize opioid receptors in the intestine, effectively reducing opioid-induced gastrointestinal dysfunction or constipation without affecting oxycodone’s analgesic effect [264]. In summary, ongoing innovation in opioid formulations and delivery methods has diversified cancer pain treatment, better addressing patients’ individual needs while reducing adverse effects and improving outcomes. 3D printing technology has gained attention in the pharmaceutical field. 3D printing enables the creation of customized medications to meet individual patient needs, especially in opioid delivery systems, where it allows precise control of drug release rates [265]. A genome-wide association study of 2057 European patients with advanced cancer receiving opioid treatment identified PCMTD2 and OPRL1 on chromosome 20 as associated with variations in the regulation of cancer pain intensity [266]. Despite issues like addiction, tolerance, and side effects, opioids remain essential for pain management. Additionally, the development of new drugs and formulations continues to refine their role in cancer pain treatment.

### NSAIDs and acetaminophen

NSAIDs relieve pain and inflammation primarily by inhibiting cyclooxygenase (COX), which reduces the production of prostaglandins from arachidonic acid [267]. Acetaminophen (paracetamol) mainly works by inhibiting CNS cyclooxygenase to relieve pain, but it has minimal anti-inflammatory effects [268]. NSAIDs are classified into three categories: selective cyclooxygenase-1(COX-1) inhibitors ( aspirin), selective cyclooxygenase-2 (COX-2) inhibitors ( meloxicam, nimesulide) and non-selective COX inhibitors ( ibuprofen, indomethacin, diclofenac) [269]. Pain management guidelines for cancer typically recommend NSAIDs and acetaminophen as first-line treatments for mild to moderate pain, especially in cases of bone metastasis, soft tissue inflammation, and related conditions. Guidelines from organizations like the WHO and NCCN recommend COX-2 inhibitors for cancer patients with liver or kidney metastases, as they may reduce the gastrointestinal side effects and renal toxicity associated with traditional NSAIDs [15, 17]. However, as NSAID dosage increases, the pain relief effect may plateau. Further increases in dosage may not improve pain relief and could significantly raise the risk of side effects [270]. Therefore, when NSAID doses approach the maximum tolerated level and pain management needs increase, switching to opioid analgesics should be considered. For patients on long-term NSAID or acetaminophen use, if the daily dose is near or exceeds the recommended maximum, further dose increases should be avoided. Instead, opioid analgesics should be used to manage pain more effectively, rather than increasing NSAID or acetaminophen doses. In combination therapy, the priority should be to increase opioid doses rather than NSAID or acetaminophen doses to minimize the risk of drug-related side effects [271, 272].

### Antidepressants

Antidepressants are particularly effective in managing neuropathic pain, especially in patients with nerve damage or cancer. These medications mainly work by modulating neurotransmitters, particularly serotonin and norepinephrine, in the brain and spinal cord, enhancing their inhibitory effects in pain pathways. Besides affecting neurotransmitters, antidepressants also alleviate emotional distress in brain regions linked to pain, which are crucial in cancer-related pain [273–275]. They are effective not only for mood disorders but also for managing cancer-related neuropathic pain [276]. Amitriptyline, a classic tricyclic antidepressant, is widely used in cancer pain management. Studies show that amitriptyline alleviates neuropathic pain by inhibiting cholinergic transmission, with notable effects in breast cancer patients [277]. However, amitriptyline may inhibit cholinesterase, limiting its use, especially in patients with cholinergic system issues, requiring caution. Nortriptyline, a metabolite of amitriptyline, offers similar pain relief with fewer side effects, making it a safer option. Nortriptyline’s analgesic effects are comparable to amitriptyline’s, particularly for neuropathic pain [278]. Duloxetine, a Food and drug administration approved antidepressant, treats depression and is highly effective in alleviating cancer-induced neuropathic pain. Studies show that duloxetine significantly reduces pain, especially in chemotherapy-induced neuropathy caused by paclitaxel [279, 280]. Consequently, duloxetine is becoming a first-line treatment for cancer pain. In contrast, venlafaxine works primarily by inhibiting norepinephrine and serotonin reuptake. It effectively alleviates peripheral neuropathic pain in cancer patients with promising results. However, dosage directly affects its efficacy: at lower doses (75 mg/day), venlafaxine mainly affects serotonin reuptake, with minimal impact on norepinephrine, resulting in milder pain relief. At higher doses (225 mg/day), it significantly affects norepinephrine reuptake, leading to stronger pain relief [281]. Although effective in managing cancer-related pain, these medications can cause side effects. Amitriptyline, nortriptyline, and duloxetine may cause dry mouth, constipation, weight gain, and drowsiness, requiring caution, particularly in elderly patients or those with heart disease [282].

### Anticonvulsants

Anticonvulsant medications are frequently used in cancer pain management, especially for neuropathic pain resulting from nerve damage. This pain is often triggered by tumor compression of nerves or by nerve injury caused by cancer treatments. Clinical guidelines commonly recommend anticonvulsants such as phenytoin, pregabalin, carbamazepine, and gabapentin. These drugs share a common mechanism of action, modulating nerve transmission to reduce excessive neuronal excitability and alleviate neuropathic pain [283, 284]. Gabapentin is widely used to treat both neuropathic pain and epilepsy. Its mechanism of action primarily involves binding to the α2-δ subunit of calcium channels, modulating neurotransmitter release and reducing abnormal signal transmission between neurons. Gabapentin stabilizes neuronal electrical activity, reduces pain signal transmission, and is extensively used in cancer-related neuropathic pain [285, 286]. Research shows that doses of 600–1200 mg of gabapentin effectively reduce mixed pain in cancer patients with mild systemic side effects [287]. The drug’s effects are especially notable when combined with opioids. A study showed that increasing the gabapentin dose from 200 to 2400 mg over 15 days significantly alleviated cancer-related neuropathic pain [288]. Additionally, combining low-dose gabapentin (400 mg/day) with a low-dose antidepressant, imipramine (10 mg/day), has proven effective in managing acute and breakthrough pain [288]. Pregabalin, with a similar molecular structure to gabapentin, acts more quickly, has higher bioavailability, and causes fewer side effects. Studies show that low doses of pregabalin, combined with low-dose antidepressants, are more effective in managing cancer pain than either treatment alone [289]. This makes pregabalin an important option for managing cancer-related neuropathic pain, especially due to growing concerns about opioid dependency and side effects. Phenytoin works by inhibiting sodium channel opening on neuronal membranes, reducing neuronal excitability, and exerting both anticonvulsant and antiepileptic effects [290]. Although less commonly used in cancer pain management, phenytoin has shown some efficacy in cases of cancer pain caused by nerve damage [291]. Lamotrigine exhibits dual therapeutic indications for seizure disorders and affective disorders by inhibiting sodium channels and enhancing gamma-aminobutyric acid (GABA) function, stabilizing neuronal membrane potentials and reducing excessive neuronal excitability. Although not a first-line choice for cancer pain management, lamotrigine has shown therapeutic benefits in certain cases of tumor-related neuropathic pain [292, 293]. In conclusion, anticonvulsants like gabapentin, pregabalin, phenytoin, and lamotrigine provide effective treatment options for cancer-related neuropathic pain, each with distinct advantages depending on the clinical scenario. The drugs selection is dictated by a constellation of clinical variables (pain type, patient response and potential side effects).

### Other analgesic medications

Cancer pain management extends beyond the conventional pharmacological treatments discussed earlier. Current clinical guidelines and evidence-based research recommend a multimodal approach to cancer pain management, incorporating medications such as local anesthetics, corticosteroids, NMDA receptor antagonists, and muscle relaxants. This approach targets different pain types through various mechanisms to improve patient comfort. Local anesthetics, like 5% lidocaine patches, are commonly used to treat neuropathic cancer pain. Lidocaine patches effectively relieve pain through local anesthetic effects, particularly for patients with localized nerve damage. This treatment has minimal side effects and is easy to use, making it ideal for patients who cannot tolerate oral or injectable medications [294, 295]. Corticosteroids, like prednisone and dexamethasone, are frequently used to manage cancer-related nociceptive pain. Corticosteroids reduce inflammation, alleviate pain by modulating the immune response, and improve appetite, energy, and overall quality of life [296, 297]. NMDA receptor antagonists, such as memantine and ketamine, are used as alternatives when cancer patients develop opioid tolerance. Memantine and ketamine inhibit NMDA receptors, block pain signal transfer in the CNS, and reduce opioid tolerance [298, 299]. These medications are typically given orally, beginning with a low dose that is gradually increased based on patient tolerance to reduce opioid dependence and manage pain. Muscle relaxants, such as baclofen and tizanidine, are also used to treat cancer-related pain due to muscle spasms. These drugs work by inhibiting nerve signal transfer in the CNS, reducing muscle tension and spasms to effectively alleviate pain. For patients with localized muscle stiffness or spasms due to cancer, muscle relaxants can significantly improve comfort [300].

## Non-pharmacological management of cancer pain

Although pharmacological treatments have been successful in alleviating cancer pain, non-pharmacological approaches have gained widespread attention as a crucial aspect of cancer pain management [301]. Numerous international and regional cancer pain management guidelines emphasize that non-pharmacological treatments not only reduce pain but also improve patients’ overall quality of life. Non-pharmacological methods are varied, including traditional physical therapies and psychological interventions including CBT, meditation, relaxation techniques, and other forms of interventional therapy [302]. The core principle of these approaches is to enhance both the physical and psychological well-being of the patient, thus alleviating cancer-related pain and associated symptoms. Non-pharmacological treatments are often combined with pharmacological therapies as adjuncts, improving pain management outcomes and the patient’s overall treatment experience (Fig. 6).

### Psychological and physical therapies

Both the WHO pain management guidelines explicitly recognize cognitive behavioral therapy (CBT) has emerged as an NCCN-endorsed psychosocial intervention for symptom modulation for alleviating chronic cancer pain and associated anxiety. In the United States, CBT is widely applied in cancer pain management and regarded as a key intervention. It helps patients reframe their cognitive patterns and emotional responses to pain, reducing pain perception and improving mood [68]. What’s more, psychological therapies are also widely used in cancer pain management [303, 304]. These treatments effectively alleviate negative emotions like anxiety and depression, helping patients reshape their pain perceptions and coping strategies, thereby improving psychological health and quality of life.

Physical therapy plays a key role in managing cancer pain. Techniques such as heat therapy, cold therapy, massage, ultrasound, and electrical stimulation effectively relieve muscle tension and localized pain in cancer patients [79, 305]. Physical therapy alleviates pain and promotes recovery by relaxing muscles, improving blood circulation, and reducing local inflammation. A study on breast cancer patients showed that three months of multidisciplinary rehabilitation significantly improved both physical function and psychological well-being, emphasizing the crucial role of physical therapy in cancer pain management and overall recovery [306]. Additionally, traditional Chinese medicine treatments have been increasingly integrated into modern cancer pain management [307, 308]. Traditional Chinese Medicine attributes cancer pain to factors such as Qi stagnation, blood stasis, and deficiencies in the liver and kidneys. Acupuncture effectively relieves pain by regulating Qi and blood flow and unblocking meridians. Tui na, through localized massage, relaxes muscles and joints, alleviating cancer-related muscle pain and joint discomfort.

### Neuroregulation technologies

Neuroregulation technologies have recently emerged as a promising approach to pain management, attracting growing attention from clinicians and researchers. These technologies are classified into two categories based on invasiveness: invasive and non-invasive. Invasive neuroregulation approaches, including spinal cord stimulation (SCS), peripheral nerve field modulation and nerve blocks, directly target the nervous system to alleviate pain effectively [309]. For instance, peripheral nerve stimulation (PNS) has shown long-term efficacy in treating chronic non-cancer pain and has shown promise in preliminary studies on cancer-related pain [310]. Non-invasive neuroregulation approaches, including transcranial magnetic stimulation (TMS) and transcranial direct current stimulation (tDCS), also show potential in pain management [311]. These methods modulate brain activity to alter pain perception, thereby reducing cancer-related pain.

## Conclusion and Perspective

Cancer pain is a common and debilitating symptom in cancer patients, severely affecting their quality of life. Research has focused on the molecular mechanisms behind cancer pain in recent years. These studies help elucidate the biological basis of cancer pain and have the potential to significantly impact clinical practice. Cancer pain development is closely linked to various molecular mechanisms, such as neuroinflammation, cytokine release, and neuronal sensitization [5, 312]. Chronic inflammation is critical for cancer progression and pain onset. Research has shown that inflammatory cytokines are key contributors to cancer pain development. Targeting these molecules could lead to novel therapeutic strategies for managing cancer pain.

Research on cancer stem cells has also revealed new insights into cancer pain. Cancer stem cells are believed to contribute to tumor resistance and recurrence. Research suggests that cancer stem cells may influence pain development by modulating signaling pathways in the tumor microenvironment. Therefore, therapeutic strategies focusing on cancer stem cells could achieve concurrent tumor regression and pain alleviation [313]. Moreover, neuroimmune interactions are pivotal in the pathogenesis of malignancy-associated pain. The interactions between neurons and immune cells enhance pain signal transmission, presenting potential targets for new pain management strategies. Modulating these interactions could improve cancer pain management [314]. Finally, pain assessment and management in clinical practice should integrate research on these molecular mechanisms. Evolving clinical frameworks increasingly position multidisciplinary models as the gold standard for neoplastic pain mitigation. In order to improve the efficacy of cancer pain management, future research should keep bridging the gap between fundamental knowledge and clinical practice.

In recent years, as our understanding of pain physiology has deepened and clinical techniques have advanced, multimodal pain management (MPM) has become an essential strategy in cancer pain management [194, 302].

Multimodal pain management involves combining various analgesic methods to target pain through different pathways and mechanisms, aiming to enhance pain relief while minimizing adverse drug effects. The primary goal involves a multimodal approach that integrates pharmacological analgesics with complementary non-pharmacological interventions, strategically engaging distinct neural pathways and molecular mechanisms to optimize therapeutic efficacy in pain modulation. MPM reduces side effects by using lower doses of various medications, thereby minimizing issues such as drug tolerance, dependence, and gastrointestinal discomfort that can result from single-drug use. Effective pain management alleviates both the physical and psychological burdens of patients, thereby improving their quality of life.

Studies show that after surgery, multimodal pain management strategies, such as combining NSAIDs, opioids, local anesthetics, and nerve blocks, could significantly reduce postoperative pain and decrease the need for opioids. This not only enhances analgesic effects but also reduces the risk of opioid dependence and misuse [315, 316]. Some studies suggest that for patients undergoing lumbar spine surgery, combining local anesthetics and NSAIDs leads to better outcomes and fewer side effects compared to using opioids alone [317, 318]. Moreover, combining opioids with gabapentin provides better pain relief and reduces side effects compared to using opioids alone [319, 320]. Multimodal pain management has also shown significant benefits in managing pain in terminal cancer patients. Research indicates that combining epidural anesthesia with systemic analgesic drugs effectively alleviates pain and boosts the living standards of these individuals [321, 322]. Additionally, incorporating CBT into pain management yields positive results. Randomized controlled trials indicate that cancer pain patients receiving CBT achieve better outcomes in pain control and emotional regulation than those relying solely on pharmacological treatments [323, 324]. In conclusion, multimodal pain management holds promising potential in cancer pain treatment. By combining pharmacological therapies, nerve blocks, psychological interventions, and other adjunctive treatments, MPM offers a comprehensive approach that significantly enhances pain relief and quality of life. Based on evidence-based recommendations, multimodal pain management is now the optimal strategy for pain management. In clinical practice. To achieve the best results, physicians must use multimodal pain treatment programs that are appropriate for each patient’s health, pain characteristics and needs.

Current cancer pain management guidelines primarily emphasize standardized pharmacological treatments but often overlook individual differences, such as cancer type, pain type, age, gender, genetic background, and comorbidities. Consequently, uniform treatment recommendations frequently fail to address the diverse needs of patients. Therefore, promoting personalized treatment strategies based on patient characteristics is crucial for increasing quality of life and efficacy [325, 326]. Moreover, cancer pain is often accompanied by psychological issues, yet existing guidelines provide limited guidance on these aspects. The interaction between psychological factors and pain is significant, as mental disorders may exacerbate pain perception. Therefore, guidelines should more comprehensively address patients’ psychological needs and provide specific strategies for psychological support to improve treatment outcomes. Some guidelines are overly theoretical and lack practical clinical guidance, making implementation challenging for healthcare providers. Therefore, clear, actionable strategies and corresponding tools should be developed to assist healthcare professionals in applying these guidelines efficiently, ensuring consistency in clinical decision-making. Effective cancer pain management relies on accurate pain assessment tools. For patients with language or cognitive impairments, flexible assessment methods, such as observational scales or family ratings, should be developed. Pain evaluation should not only quantify pain intensity but also consider emotional status, functional levels, and social support for more precise management. Overall, cancer pain management requires several improvements based on existing guidelines. These improvements include promoting personalized treatments, optimizing psychological support, refining clinical guidance, and improving assessment tools to better meet the diverse patient’s needs and enhance treatment outcomes.

Cancer pain management is a complex and multidimensional field. Future research and clinical practice should prioritize personalized treatment, further investigate molecular mechanisms, and enhance interdisciplinary collaboration. The continuous emergence of new drugs and therapies will likely lead to more effective cancer pain management strategies, ultimately improving patients’ quality of life. Achieving this goal requires increased investment and support in cancer pain management, coupled with continuous research, education, and training to foster innovation and development.

## Data Availability

Not applicable.
